# Spotlight on protein N-terminal acetylation

**DOI:** 10.1038/s12276-018-0116-z

**Published:** 2018-07-27

**Authors:** Rasmus Ree, Sylvia Varland, Thomas Arnesen

**Affiliations:** 1 Department of Biological Sciences, University of Bergen, Thormøhlensgate 55, N-5020 Bergen, Norway; 2 Department of Biomedicine, University of Bergen, Jonas Lies vei 91, N-5020 Bergen, Norway; 3 Department of Surgery, Haukeland University Hospital, N-5021 Bergen, Norway; 4 Terrence Donnelly Center for Cellular and Biomolecular Research, University of Toronto, 160 College Street, Toronto, ON M5S 3E1 Canada

**Keywords:** Protein folding, Protein folding

## Abstract

N-terminal acetylation (Nt-acetylation) is a widespread protein modification among eukaryotes and prokaryotes alike. By appending an acetyl group to the N-terminal amino group, the charge, hydrophobicity, and size of the N-terminus is altered in an irreversible manner. This alteration has implications for the lifespan, folding characteristics and binding properties of the acetylated protein. The enzymatic machinery responsible for Nt-acetylation has been largely described, but significant knowledge gaps remain. In this review, we provide an overview of eukaryotic N-terminal acetyltransferases (NATs) and the impact of Nt-acetylation. We also discuss other functions of known NATs and outline methods for studying Nt-acetylation.

## Protein modification: A small change with big effects

A chemical modification of protein structure called N-terminal acetylation occurs normally in many circumstances, but is also implicated in diseases including cancers and developmental disorders. It adds an acetyl group, composed of a carbonyl group (C = O) linked to a methyl group (CH_3_), to the nitrogen atom found at one end of a protein chain. Thomas Arnesen at the University of Bergen in Norway and colleagues review the understanding of this modification and survey the enzymes that carry it out. Acetylation occurs on around 80% of human proteins and affects crucial aspects of their function. These include the stability that determines proteins’ lifetimes inside cells, the three-dimensional structures that proteins fold into, and the interactions between different proteins. Advances in analytical techniques are yielding new insights into the role of N-terminal acetylation in health and disease.

## Introduction

Proteins embark on a cellular journey toward maturity through a diversity of molecular interactions. The maturation process begins during protein synthesis when the nascent polypeptide chain sends a signal to the ribosomal exit tunnel to balance translational speed with folding pathways, which affects protein processing, targeting, and function^1,2^. A repertoire of ribosome-associated protein biogenesis factors orchestrates these events. Consequently, the ribosomal landscape is dynamically changing in time and space to ensure the correct processing of nascent chains as they emerge from the exit tunnel^3^. N-terminal acetyltransferases (NATs) are prominent players in co-translational protein maturation, affecting the N-terminal extremity of most proteins physiochemically. On the other hand, post-translational N-terminal acetylation (Nt-acetylation) and NATs acting from the ribosome are poorly understood. Nt-acetylation and NATs have been implicated in several diseases, including cancers^4^ and developmental disorders^5–9^. The roles of NATs in development and disease are reviewed in other articles in this issue^10–13^. In this review, we provide an overview of Nt-acetylation and highlight how this modification affects protein fate in eukaryotic cells. Furthermore, we summarize and discuss methods to decipher this prevalent protein modification.

## NT-acetylation: what, how and when

### Acetylation of proteins

**Fig. 1:**
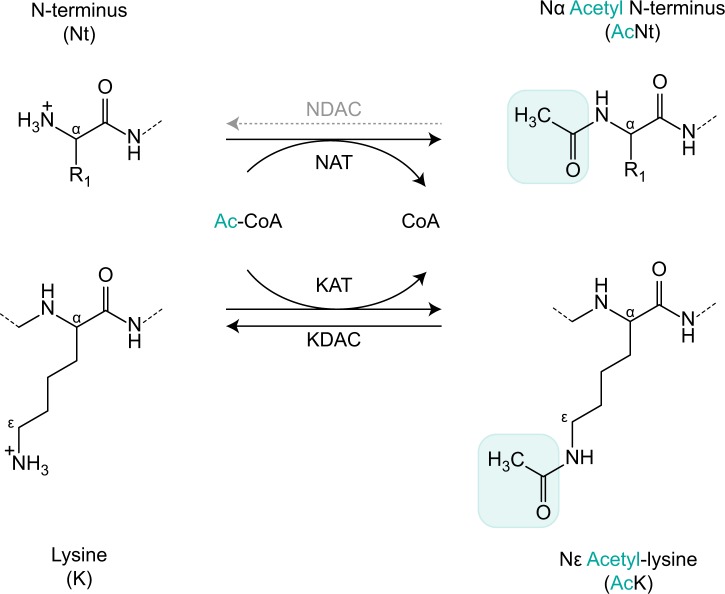
Schematic outline of N-terminal and lysine protein acetylation. N-terminal acetyltransferases (NATs) and lysine acetyltransferases (KATs) catalyze the transfer of an acetyl group (CH_3_O, turquoise) from acetyl-CoA (Ac-CoA) to the free α-amino group of protein N-termini and to the ε-amino group of lysine (K) side chains, respectively. Covalent attachment of an acetyl group eliminates the positive charge (+) of the amino group, thus affecting local electrostatic properties. Nt-acetylation is considered irreversible because an N-terminal deacetyltransferase (NDAC) remains to be discovered. In the case of lysine acetylation, the acetyl moiety may be removed by lysine deacetyltransferases (KDACs), making it a reversible protein modification

Protein Nt-acetylation refers to the covalent attachment of an acetyl group (CH_3_CO) to the free α-amino group (NH_3_^+^) at the N-terminal end of a polypeptide (Fig. 1)^14^. Protein acetylation also frequently occurs on the ε-amino group of lysine side chains^15^, which is catalyzed by lysine acetyltransferases (KATs) (Fig. 1). The deacetylation reaction is catalyzed by lysine deacetylases (KDACs); corresponding N-terminal deacetylases (NDACs) have not been discovered, thus Nt-acetylation is considered irreversible. By neutralizing the positive charge of the N-terminus, this widespread protein modification greatly affects the electrostatic properties of proteins and would be expected to modify protein function. Indeed, irreversible Nt-acetylation impacts a wide range of protein properties, including stability^6,16–19^, folding^20–22^, protein–protein interactions (PPIs)^23–28^, and subcellular targeting^29–32^. A number of biological processes are thereby steered by Nt-acetylation, including an emerging role in transcriptional control through histone tail modifications^33,34^.Thus, the chances are high that your favorite protein is acetylated at the N-terminus and possibly contains multiple sites of lysine acetylation. The molecular mechanism and functional consequences of reversible lysine acetylation are addressed in other articles within this issue^10–12,35^. For a comprehensive review on protein acetylation, the reader is referred to Drazic et al.^36^ and the references therein.

### NAT composition and specificity

Nt-acetylation is catalyzed by highly conserved NAT enzymes, which differ from each other with respect to their subunit composition and substrate specificity profiles. The majority of eukaryotic Nt-acetylation reactions are achieved through oligomeric complexes NatA, NatB, and NatC, which consist of at least a unique catalytic subunit and one unique ribosomal anchor that contributes to substrate specificity and interacts with nascent polypeptides^37^. The substrate specificities of NAT enzymes are mainly determined by the identities of the first two N-terminal residues. The human NatA complex, which consists of the catalytic subunit NAA10, the ribosomal anchor NAA15 and the auxiliary subunit HYPK, co-translationally acetylates N-termini that bear a small amino acid (A, S, T, C, and occasionally V and G), which is exposed after methionine cleavage by methionine aminopeptidases (MetAPs)^38–42^. Notably, NAA10 also exists in a monomeric state and can post-translationally acetylate acidic N-termini (D-, E-) in vitro, but it does not appear to have activity towards classical NatA-type substrates^39,43^. NatB and NatC acetylate N-terminal methionine with further specificity determined by the identity of the second amino acid. The NatB complex is formed by the catalytic subunit NAA20 and the ribosomal anchor NAA25 and acetylates methionine-acidic/hydrophilic N-termini (MD-, MN-, ME-, and MQ-)^44,45^. The NatC complex comprises three subunits: the catalytic component NAA30, the ribosomal anchor NAA35, and the auxiliary subunit NAA38, whose precise role has not been determined. Human NatC acetylates proteins with hydrophobic/amphipathic N-termini (ML- MI-, MF- MW-, MV-, MM-, MH-, and MK-) to varying degrees^46,47^. Naa40 (NatD) is on the other hand, a highly selective NAT that specifically Nt-acetylates histones H2A and H4 (S-G- starting)^48,49^. NAA50 physically interacts with the NatA complex, but it displays distinct enzymatic activity and is termed NatE^50–54^. The in vitro substrate specificity profile of NatE partly overlaps with NatC, thus implying a potential functional redundancy. Studies performed in *S. cerevisiae* revealed that human NAA50 can potentially Nt-acetylate initiator methionine followed by a small amino acid residue, and consequently, it can cause differential initiator methionine processing^53^. However, these N-termini are typically processed by MetAPs and Nt-acetylated by NatA. Since a myriad of reactions take place in the vicinity of the ribosomal exit tunnel, the activities of NAA50, NatA, and MetAPs are most likely well-coordinated, but the functional implications of this molecular interplay are currently not known. NAA50 might also have functions that are independent of NatA and the ribosome. The classical view of Nt-acetylation almost exclusively occurring during protein synthesis was recently challenged by the identification of NAA60. NAA60 (NatF) is an organellar NAT that is anchored to the cytosolic side of the Golgi membrane, where it specifically acts on transmembrane proteins^55–57^. The substrate specificity of NatF partly overlaps with NatC and NatE (Table 1); however, considering that these NATs are most likely exposed to distinctive substrate pools within the cell, their functional redundancy might be minor. Furthermore, in the plant kingdom, the lumenal chloroplastic NAT NAA70 was also recently discovered^58^.

**Table 1:** Composition and substrate specificity of human NATs

	NatA	NatB	NatC	NatD	NatE	NatF^b^
Catalytic subunit	NAA10 (ARD1)	NAA20 (NAT3)	NAA30 (MAK3)	NAA40 (NAT4)	NAA50 (NAT5, SAN)	NAA60
Auxiliary subunit	NAA15 (NATH/NAT1) HYPK	NAA25 (MDM20)	NAA35 (MAK10) NAA38 (MAK31)	—	NAA10 NAA15	—
Specificity^a^	A-	MD-	ML-	S-G-G-(H2A and H4 histones)	MK-	MK-
	S-	MN-	MI-		MV-	MS-
	T-	ME-	MF-		MA-	MV-
	C-	MQ-	MW-		MY-	ML-
	V-		MV-		MF-	MQ-
	G-		MM-		ML-	MI-
			MH-		MS-	MY-
			MK-		MT-	MT-
Loss of function phenotypes in human cells	Apoptosis Cell cycle arrest	Cell cycle arrest	Apoptosis Mitochondrial defects Golgi fragmentation	Apoptosis Loss of mesenchymal phenotype	Sister chromatid cohesion and chromosome condensation	Golgi fragmentation
Key references	^38,39,41,42,99^	^44,45,100,101^	^46,47,102^	^33,34,49,103^	^50–53,104,105^	^55–57,84^

^a^Amino acids are listed according to in vivo Nt-acetylation specificity

^b^NatF resides on the Golgi membrane, where it most likely performs post-translational Nt-acetylation^56^. Commonly used alternative names are given in parentheses

### Introducing the N-terminal acetylome

**Fig. 2:**
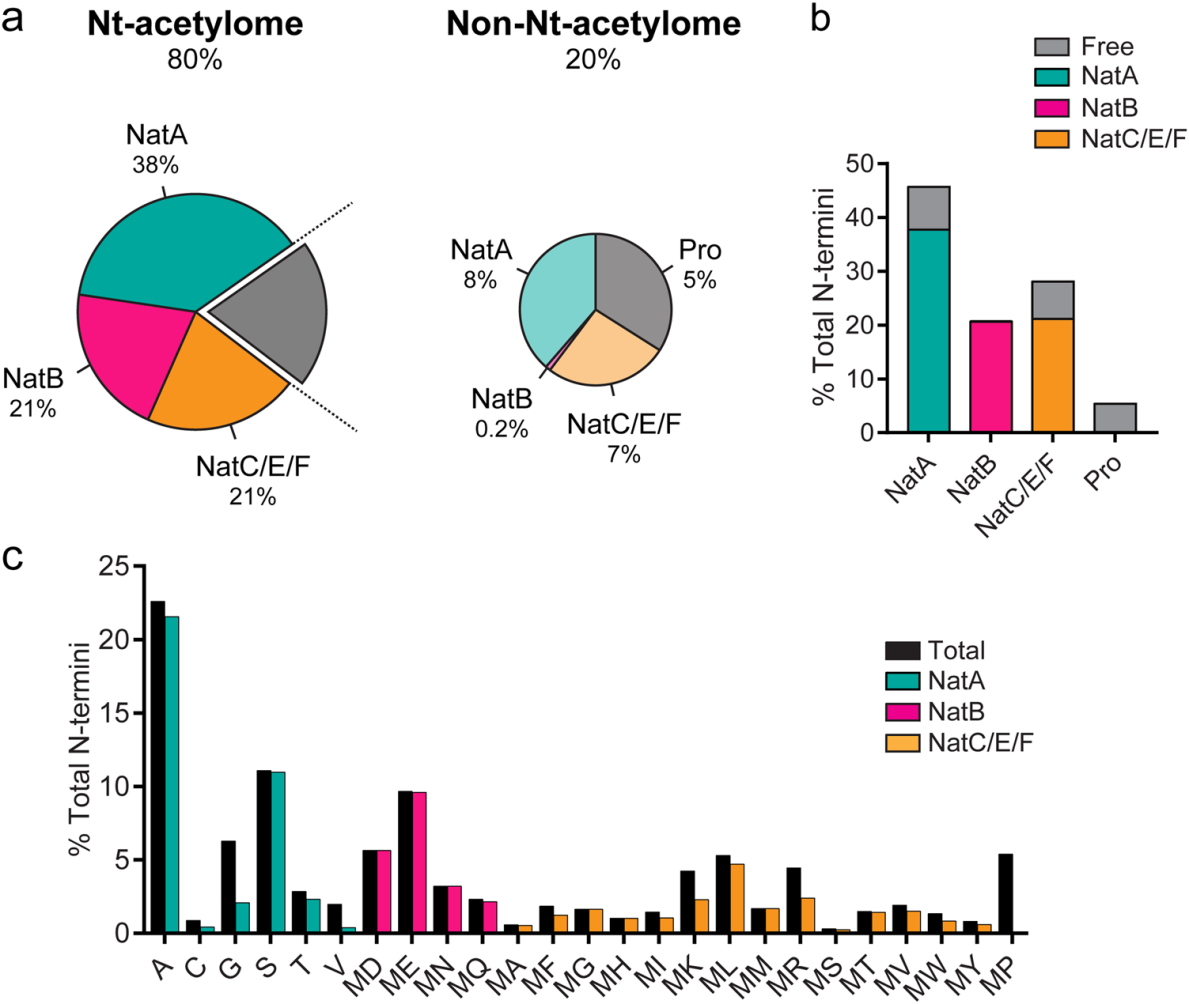
Mapping the human Nt-acetylome. **a** The prevalence of Nt-acetylation in human cells is visualized by separating the proteome into the Nt-acetylome (80%) and the non-Nt-acetylome (20%). The human Nt-acetylome was predicted by incorporating experimentally determined Nt-acetylation events (including NatC-^47^ and NatF-^56^related data) to all SwissProt entries (version 57.8) based on the occurrence of the first two amino acids. Note that this global estimate does not consider the distinction between full and partial Nt-acetylation. Thus, a given protein can exist in both Nt-acetylated and non-Nt-acetylated forms. The chance that your favorite protein will be Nt-acetylated mainly depends on the identity of the first two amino acids. To visualize this concept, the Nt-acetylome can be grouped according to **b** NAT substrate class and/or **c** N-terminal amino acid frequency. For example, NatB-type substrates (MD-, ME-, MN- MQ-) are almost fully Nt-acetylated and account for 21% of the proteome. The combined NatC/E/F substrate class needs further refinement, which is reflected by a coverage rate of 75%. For example, an estimated 89% of ML-starting N-termini are Nt-acetylated, which is in stark contrast to MK- starting N-termini, where only 54% of N-termini are thought to be Nt-acetylated. MW- and MR- data are inferred based on structural similarity to MF- and MK-, respectively. NatD is not depicted due to its limited coverage

Nt-acetylation is a common protein modification, affecting an estimated 80% of all human protein species to a varying extent^37,41,55,56^. The N-terminal acetylome (Fig. 2a), referring to the complete set of Nt-acetylated proteins, was deduced by extrapolating experimental data from proteomic studies to the human proteome^37,56,59^. The relative contribution of individual NATs to the Nt-acetylome can be inferred by clustering the proteome into NAT substrate classes (Fig. 2b). The NatA class (green) accounts for 46% of the proteome, where 83% is Nt-acetylated and 17% is unacetylated. Thus, NatA is estimated to Nt-acetylate 38% of the proteome. The NatB class (pink) covers 21% of the proteome, and almost every protein of this type is Nt-acetylated. The combined NatC, E and F substrate class (orange) accounts for 28% of the proteome, but with nearly 7% of the proteins being unacetylated, it only has 75% coverage. The acetylation status of protein N-termini is mainly determined by the identity of the first two amino acids (Fig. 2c). For example, the likelihood of a NatC/E/F-type substrate being Nt-acetylated ranges from 100% (MM-) to 54% (MK-). Further, an acidic residue (D/E) in position two promotes Nt-acetylation (>95%), whereas a proline residue (P) in the second or first position prevents Nt-acetylation, thus ensuring free N-termini. The latter is referred to as the (X)PX rule and is often used in functional studies of Nt-acetylation^60^.

Currently, the human Nt-acetylome is based on proteomics surveys and extrapolated to the rest of the proteome by the identity of N-terminally acetylated amino acids. It does not consider N-terminal topologies and barely reflects whether NATs are exposed to confined parts of the proteome. NatF, for example, resides on the Golgi and has selectivity toward transmembrane proteins^56^. Differences in substrate availability for NATs may explain why some proteins are fully acetylated, whereas others are partially Nt-acetylated; thus, proteins exist in both acetylated and unacetylated forms. Furthermore, the NAT machinery apparently did not diversify during the evolution of eukaryotes despite the increased complexity of their proteome. Based on this observation, it has been suggested that NAT activity is largely regulated through transcriptional mechanisms and post-translational modifications^61^. We therefore anticipate further refinement of Nt-acetylome estimates and NAT substrate specificity in the future.

## The fate of NT-acetylated proteins

The cellular fate of Nt-acetylated proteins is as multifaceted as the proteins receiving the acetyl group. The molecular effects of Nt-acetylation are highly dependent on the substrate and its cellular context, but some roles have been discovered. Here, we highlight some protein-specific consequences of Nt-acetylation, which range from protein lifespan alterations to subcellular localization changes, that were discovered within the last decade.

### Protein half-life regulation and the Ac/N-end rule pathway

**Fig. 3:**
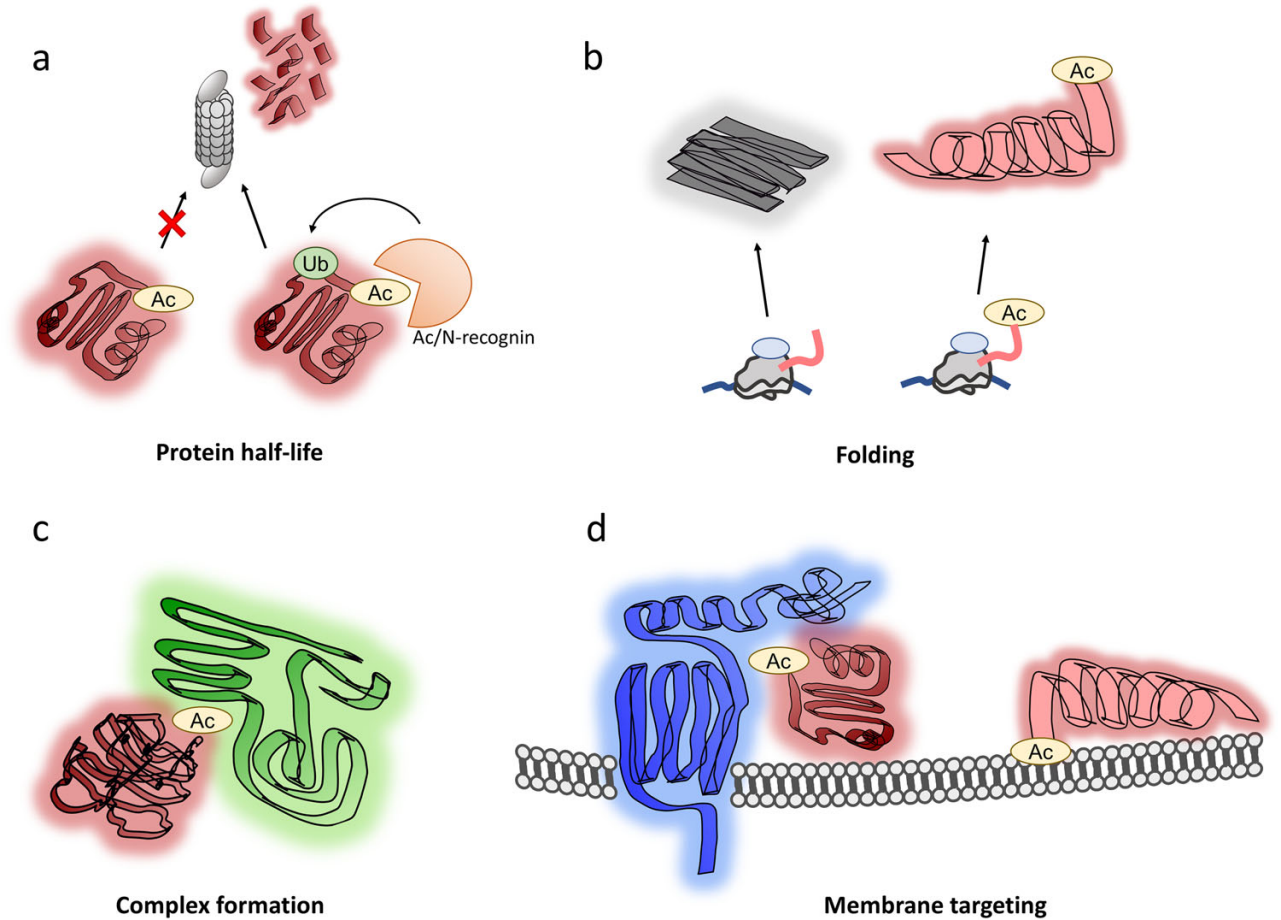
Functions of N-terminal acetylation. Nt-acetylation has many functions in the cell. **a** In some cases, Nt-acetylation targets proteins for polyubiquitination and proteasomal degradation, and in other cases, it protects against such degradation. **b** Nt-acetylation is required for the proper folding of some proteins. **c** Protein–protein interactions (PPIs) in protein complex formation are in some cases mediated or augmented by Nt-acetylation. **d** Nt-acetylation serves to target some proteins for membranes, either through PPIs with an integral membrane protein or through direct interactions with membrane lipids. Ac acetyl group, Ub ubiquitin

The perhaps most discussed function of Nt-acetylation is the targeting of acetylated proteins for polyubiquitination and degradation by the proteasome as part of the Ac/N-end rule^62^. The Ac/N-end rule is reviewed more thoroughly in another article in this issue^63^. The Nt-acetyl group was first described as a specific degradation signal in 2010, when it was shown that the yeast E3 ligase Doa10 can target proteins with an acetylated N-terminal methionine, alanine, valine, serine, threonine, or cysteine^16^. This targeting is manifested as reduced protein half-life (Fig. 3a). It is apparent, however, that the Ac/N-end rule is sensitive to the environment of the Nt-acetylated protein. When the yeast protein Cog1 is Nt-acetylated, the resulting Ac/N-degron is recognized by the Ac/N-recognin Not4 and subsequently degraded. However, the Cog1 Ac/N-degron is conditional, and may be shielded by interaction partners (in the case of Cog1, these partners are other subunits of the COG complex). When Cog1 and its interactors are present in an even stoichiometry, this shielding is complete, whereas overexpressed Cog1 is unshielded and prone to degradation. In this simple and elegant way, the cell can regulate the relative levels of protein complex subunits^17^. The importance of context dependence is also made apparent by the presence of several substrates that are stabilized by Nt-acetylation. Loss of Nt-acetylation leads to the rapid proteasomal degradation of THO complex subunit 7 homolog in human cells^6^ and of an *Arabidopsis thaliana* Nod-like receptor (NLR). Alternative translation initiation sites in the mRNA encoding NLR Snc1 yield a protein with the N-terminal sequence MMD (surprisingly, this protein is reported as a NatA substrate in this paper^18^) or MD (a NatB substrate). Contrary to the Ac/N-end rule, the proteoform with the N-terminus Ac-MMD (canonically, a stabilizing combination^62^) is degraded, whereas the Ac-MD form (nominally a destabilizing N-terminus) is stable^18^. A similar case is found with methyl CpG-binding protein 2 (MeCP2). An Ala2Val mutation in MeCP2 causes Rett syndrome. Both the Ala- and Val forms are subject to methionine excision and Nt-acetylation, but only Ac-Val-MeCP2 is unstable, suggesting that Nt-acetylation of MeCP2 is stabilizing but dependent on the N-terminal amino acid^19^ (Fig. 3a). This finding also represents an interesting case in which the loss of Nt-acetylation and subsequent premature degradation are associated with a developmental disorder.

### Nt-acetylation and protein folding

Nt-acetylation is implicated in protein quality control and protein folding (Fig. 3b). Checkpoint kinase 1 (Chk1) has a significantly shortened half-life in *naa10*Δ yeast, owing to its proteasomal degradation through the Arg/N-end rule pathway, a branch of the N-end rule pathway that is distinct from the Ac/N-end rule^64^. Although the mechanism behind this effect is not yet clear, NatA is necessary for the interaction of Chk1 and its chaperone, a heat shock protein (Hsp) 90 subunit. Notably, this chaperone activity is necessary for the stability of Chk1. Deletion of naa10 led to the downregulation of Hsp90 components, whereas the Arg/N-end degradation pathway displayed increased activity^64^. In another study, NatA was essential for proper Hsp70 function, and extensive protein misfolding was observed in a NatAΔ strain, thus pointing to a general role for Nt-acetylation in chaperone-assisted folding^22^. For some human substrates, the link between aggregation and disease phenotypes is suggested, although no causal relationships have been established. In the case of α-synuclein, NatB Nt-acetylates it to stabilize an N-terminal α-helix, increasing its resistance to aggregation^20,65^. This finding may have implications for the development of Parkinson’s disease, as the aggregation of α-synuclein is one of its primary hallmarks. Depletion of the NatA components NAA10 or HYPK leads to huntingtin aggregation, connecting it to pathogenesis of another neurodegenerative disorder^42^.

### Nt-acetylation mediates protein complex formation

When the N-terminus is acetylated, the altered charge state and increased hydrophobicity may create a new protein interaction surface (Fig. 3c). This effect allows the formation of the E2/E3 complex Ubc12/Dcn1. The Nt-acetylated iMet of Ubc12 is docked within a hydrophobic pocket of Dcn1, promoting the functional role of the E2/E3 complex in a conserved manner^24,25^. Nt-acetylation also stabilizes an interaction with Sir3 to nucleosomes by inducing a conformational shift in a loop in Sir3 that is responsible for binding the nucleosome core particle^26,27^.

### Nt-acetylation mediates membrane targeting and subcellular localization

Nt-acetylation was shown to direct proteins to membranes through two different mechanisms (Fig. 3d). One mechanism is through an association with an integral membrane protein, as is the case for the peripheral Golgi protein Arl3, which is anchored to the membrane by Sys1^29,30^. The other mechanism is through direct membrane interaction, as is the case for α-synuclein. The Nt-acetylation of α-synuclein stabilizes the terminal α-helix and increases its affinity for moderately charged vesicles^66^.

### The emerging role of Nt-acetylation as a histone modification

**Fig. 4:**
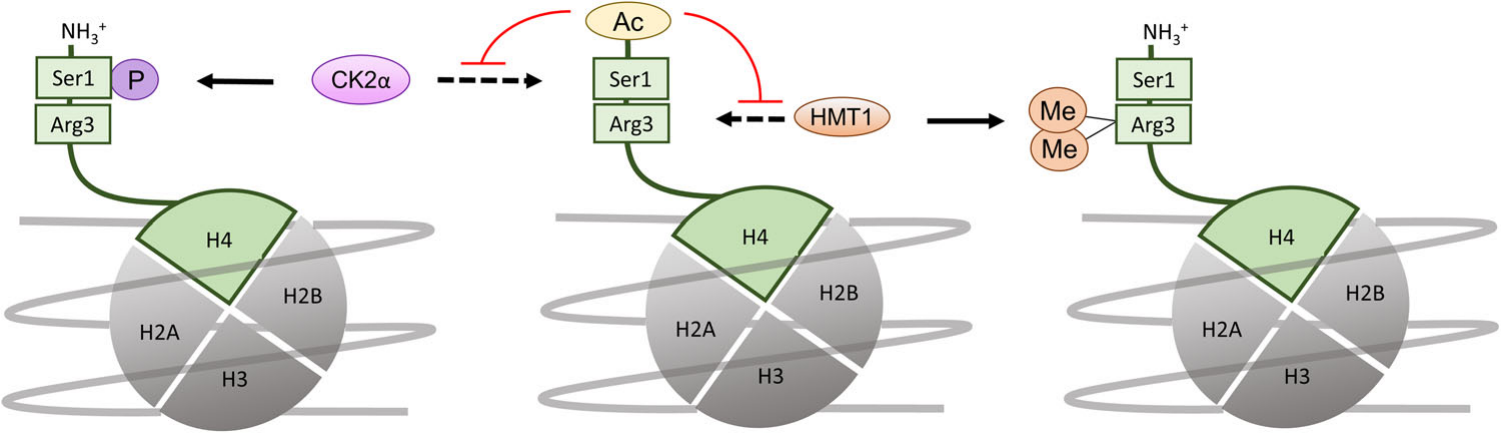
Crosstalk between Nt-acetylation and other modifications near the histone H4 N-terminus. Nt-acetylation of histone H4 precludes other modifications near the N-terminus. Nt-acetylation of H4 blocks the nuclear translocation of casein kinase 2, α subunit (CK2α) and phosphorylation of Ser1 on H4. Arg3 dimethylation of yeast H4 by histone methyltransferase 1 (HMT1) is also precluded by H4 Nt-acetylation

NatD (NAA40) is perhaps the most specialized known NAT, having only two substrates: histones H2A and H4^48,49^. H4 is subject to methionine excision, and the serine in the first position is Nt-acetylated by NatD. This modification affects gene expression patterns in interesting ways and has implications for cancer progression and lifespan, as the Nt-acetyl group of H4 was shown to preclude other H4 histone tail modifications (Fig. 4). The transcription factor Slug participates in the epithelial-mesenchymal transition, a key event in cancer cell invasion and metastasis. Nt-acetylation of H4 blocks the nuclear translocation of casein kinase 2, α subunit (CK2α), its binding to H4, and the subsequent phosphorylation of Ser1 on H4. This block promotes transcription of Slug and a mesenchymal phenotype, boosting the metastatic potential of Slug-transcribing cells^34^. The same acetylation event is responsible for blocking the dimethylation of Arg3 on H4 in yeast^33,67^. Crosstalk between Nt-acetylation and other modifications at or near the N-terminus thus impacts cell fate and has implications for aging and carcinogenesis. Furthermore, it establishes NatD as a chromatin modifier. It will be interesting to see whether the Nt-acetylation of H4 happens exclusively at the ribosome, or whether it may also take place post-translationally in the nucleus. While the Nt-acetylation of H2A and H4 has been considered a co-translational reaction, the fact that a significant fraction of NatD is not associated with the ribosome, including a portion found in the nucleus^49^, suggests that NatD may have a role here. The prevalence of H4 Nt-acetylation in human cells should also be clarified (it is ~100% in yeast^49^). The H4 N-terminus is an epigenetic mark that currently appears to be unregulated and irreversible, like other Nt-acetylation events. However, it responds to changes in NatD levels^34^.

## NATs as multifunctional enzymes?

**Fig. 5:**
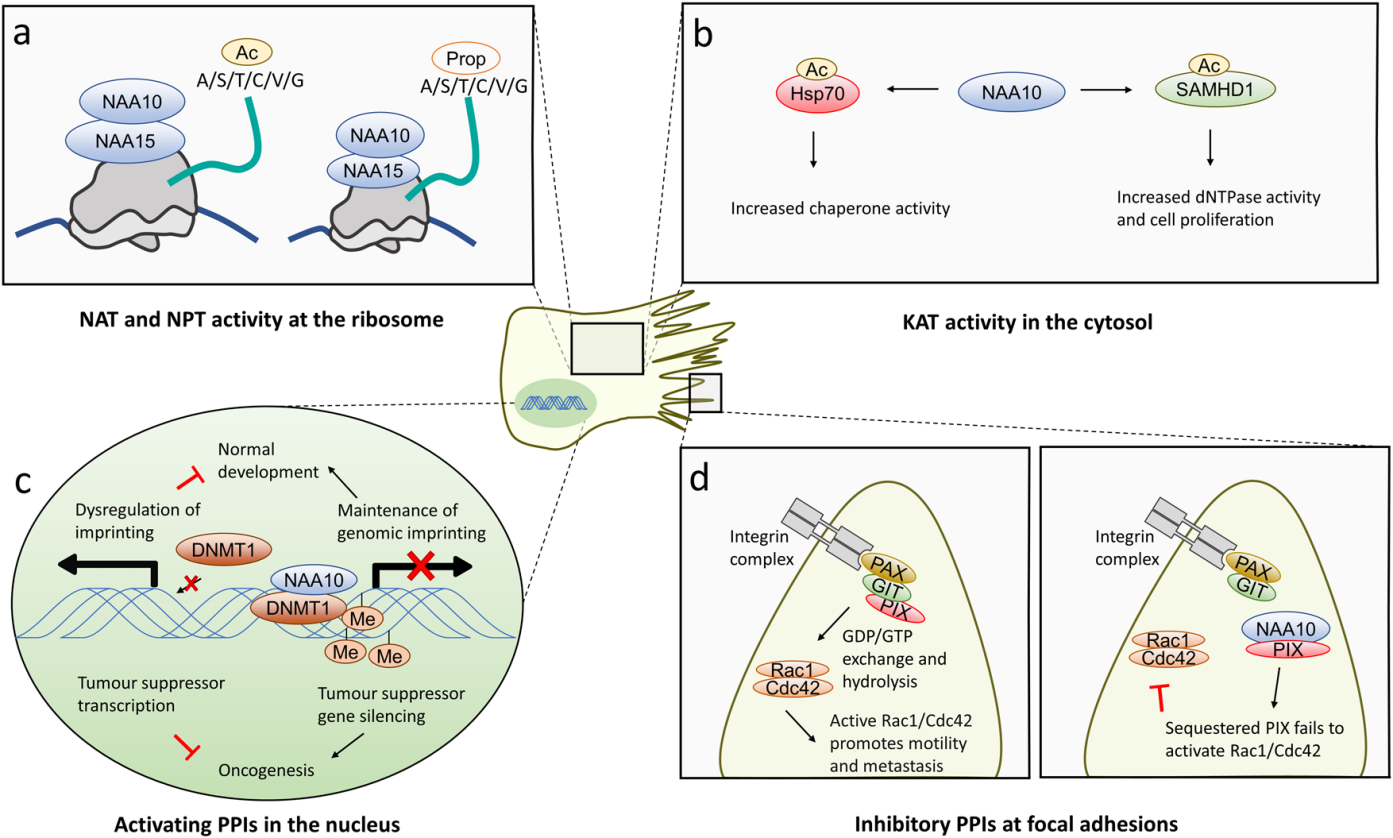
Functions of NAA10. **a** NAA10 functions as a cotranslational NAT as part of the NatA complex on the ribosome, acetylating small or polar amino acids (alanine, serine, threonine, cysteine, valine, or glycine). It also has N-terminal propionyltransferase (NPT) activity toward the same substrates as for its NAT activity, transferring a propionyl group (Prop) to the N-terminus, albeit at a significantly lower frequency than Nt-acetylation. **b** As a lysine acetyltransferase (KAT), NAA10 acetylates Hsp70, causing it to shift towards chaperone activity. It acetylates SAMHD1, enhancing its dNTPase activity and promoting cancer cell proliferation. **c** NAA10 binds DNMT1 and mediates its interaction with DNA. DNMT1 then imprints DNA. Loss/depletion of NAA10 leads to dysregulation of this imprinting and inhibits normal development, but it may also suppress oncogenesis. **d** NAA10 interaction with PIX prevents the formation of the GIT/PIX/paxillin complex at focal adhesions, inhibiting GDP/GTP exchange on Rac1/Cdc42 and subsequent cell motility. NAT N-terminal acetyltransferase, KAT lysine acetyltransferase, NPT N-terminal propionyltransferase, A alanine, S serine, T threonine, C cysteine, V valine, G glycine, Ac acetyl group, Prop propionyl group, NAA N-α-acetyltransferase, SAMHD1 SAM domain and HD domain containing protein 1, Hsp70 Heat shock protein 70, PPI protein–protein interaction, DNMT1 DNA methyltransferase 1, Me methyl group, PAX paxillin, GIT ARF GTPase-activating protein GIT1, PIX PAK-interacting exchange factor, Rac1 Ras-related C3 botulinum toxin substrate 1, Cdc42 cell division control protein 42

The first described molecular function of NAA10 was its NAT function as the catalytic subunit of the NatA complex with its partner NAA15^38,40^, and this function is evolutionarily conserved^41^. This activity also extends to other Nt-acylations, including the use of propionyl-CoA for Nt-propionylation, although this modification is less frequent than Nt-acetylation (Fig. 5a)^68^. In addition to thousands of unique cellular substrates for its NAT activity^41^, there are numerous reports that NAA10 also has KAT activity^69–77^, and some publications also point to acetyltransferase-independent functions^78–80^. Through its proposed KAT activity, NAA10 is reported to impact substrates at the central junctions of cell signaling networks (Fig. 5b). In this manner, NAA10 is suggested to control or fine-tune processes such as cell division, apoptosis and the cellular stress response. The activity of 70 kDa heat shock protein (Hsp70), a molecular chaperone that is upregulated in stress conditions, increases after Naa10-catalyzed acetylation^70^. SAM domain and HD domain containing protein 1 (SAMHD1) is acetylated on an internal lysine by NAA10, upregulating the dNTPase activity of SAMHD1 and promoting progression through the cell cycle^71^.

Some reported lysine substrates of NAA10 have failed replication, however. Marmorstein and colleagues performed systematic in vitro KAT assays using several reported NAA10 KAT substrates and could find no evidence of NAA10-catalyzed lysine acetylation^81^. Testing the activity of NAA10 towards the reported substrates Runt-related transcription factor 2^75^, methionine sulfoxide reductase A^77^, and myosin light-chain kinase^76^, they found that representative peptides and whole substrates were abundantly chemically acetylated independent of the presence of NAA10^81^. Another purported NAA10 KAT substrate, β-catenin, was likewise not replicated. NatA depletion in CAL-62 and 8350C cells did not have any effect on β-catenin acetylation, suggesting that β-catenin is acetylated by a different mechanism^82^. The crystal structure of NAA10 has a loop that extends over the substrate binding site, restricting access to the active site to N-terminal amine substrates while excluding lysine substrates^39,81^. A corresponding loop is present in all human NAT structures that have been determined to date, including NAA40^83^, NAA50^52^, and NAA60^84^.

All in all, the present evidence that NAA10 has limited KAT activity is compelling. The available structural evidence speaks against this activity, several published instances of in vitro lysine acetylation can be explained by chemical acetylation due to high concentrations of acetyl-CoA, and the pleiotropic effects of NAA10 depletion may be sufficient to explain the observed phenotypes. However, we also know that NAA10 switches its substrate specificity after NatA complex formation-induced conformational changes^39,43^. Furthermore, a significant fraction of NAA10 is present outside of the NatA complex^43^, and NAA10 is often found in the nucleus^38,85^ and has interaction partners that are not part of the NAT machinery at the ribosome^78–80^. Thus, it cannot be discounted that factors exist that mediate a shift from NAA10’s NAT- to KAT activity.

In addition to its well-established acetyltransferase activity, there is evidence that NAA10 can influence gene expression patterns and cell motility through protein interactions. Findings from yeast two-hybrid assays and chromatin immunoprecipitation connect NAA10 to DNA methylation-dependent gene repression. DNA-methyltransferase 1 (DNMT1) is recruited by NAA10 to the promoters of tumor suppressor genes, methylating them and blocking their expression (Fig. 5c). Crucially, this happens in an acetyltransferase-independent manner^78^. A recently published *Naa10* KO mouse model also had globally reduced DNA methylation and concomitant increases in gene expression^79^.

NAA10 is also reported to have a protein/protein interaction-mediated impact on cell motility. The GIT/PIX/paxillin complex localizes to focal adhesions, where it regulates cytoskeletal dynamics and cell migration through the activation of Cdc42/Rac1. When NAA10 binds to PIX, the GIT/PIX interaction is precluded, inhibiting cell motility^80^ (Fig. 5d).

## Techniques for studying NT-acetylation

**Fig. 6:**
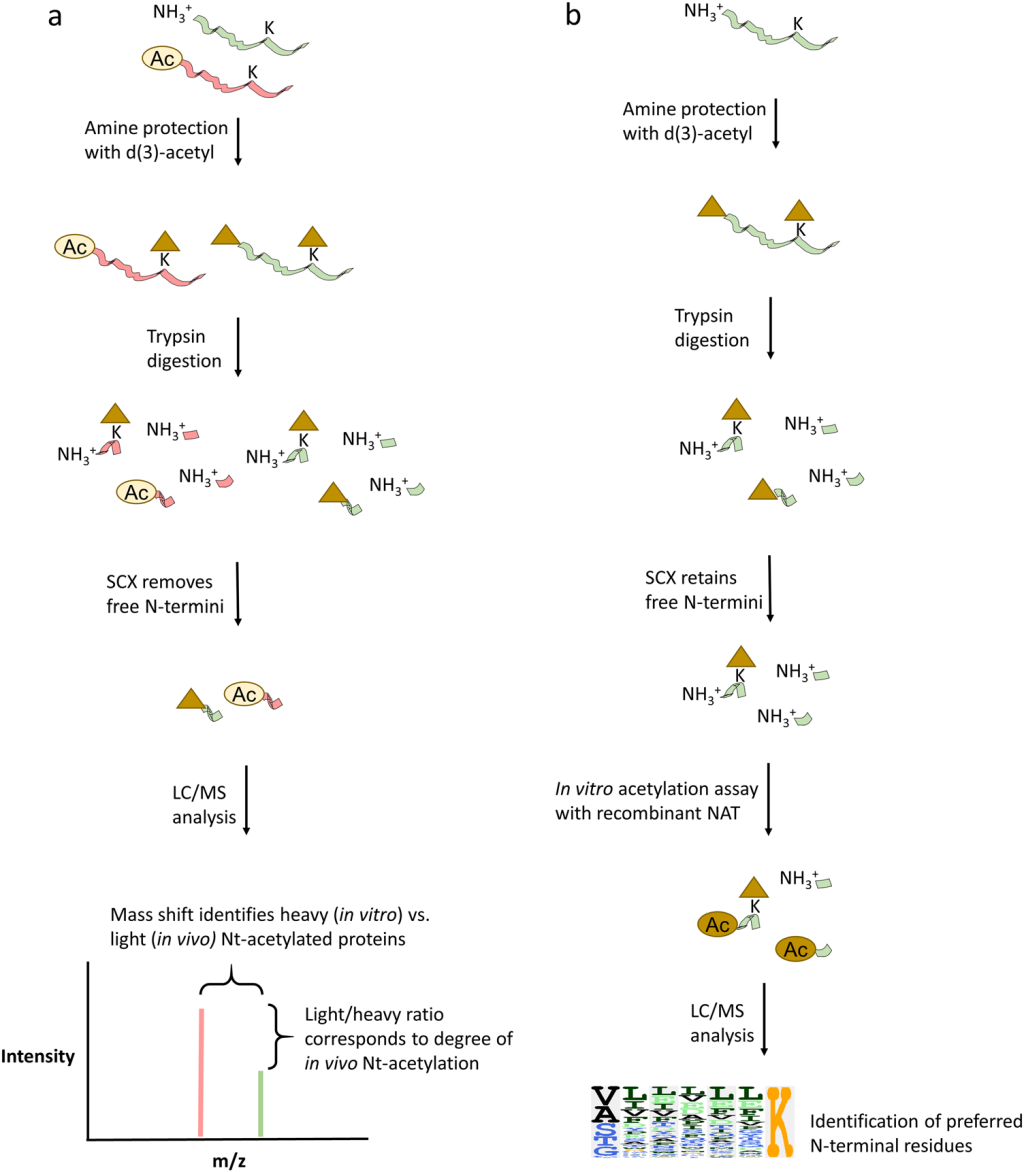
MS-based approaches for the identification of NAT substrates and substrate specificities. **a** The main steps of the workflow for SILProNAQ and COFRADIC. A protein mixture is trideutero-acetylated on free amine groups (N-termini or lysine residues (K)), digested with trypsin, and solid cation exchange (SCX) fractionated to remove free N-termini. In COFRADIC, a two-step fractionation scheme is performed at this stage (not shown). Peptides are then quantified by LC/MS, and the magnitude of intensity change in light/heavy Nt-acetyl groups (non-deuterated vs. trideuterated Nt-acetyl) measures the fraction of acetylation for a given N-terminus. **b** The generation of peptides for an in vitro peptide library is performed in the same manner as that of COFRADIC/SILProNAQ, except that at the SCX stage, peptides with free N-termini are retained rather than being discarded and are used as substrates for recombinant NAT enzymes. The output of this reaction is measured by LC/MS and is used to deduce the substrate preference of the NAT in question

Usually, the lysine acetylome is characterized and quantified by enrichment with antibodies against acetylated lysine residues, which is followed by mass spectrometric analysis^86–88^. This enrichment enables the quantification of acetylated peptides, which are often scarce due to the low stoichiometry of many Ac-lysine species^89^ and the fact that only one relevant peptide can exist per protein molecule. Although Nt-acetyl stoichiometry is usually much higher than that of Ac-lysine, there are many internal peptides for each N-terminal peptide, so proteomic techniques must have a way of enriching the N-terminal peptides under examination (Fig. 6). This enrichment is typically done in two steps. First, unprotected amines are chemically acetylated with heavy acetyl (either trideutero- or ^13^C_2_-labeled) to distinguish between in vitro and in vivo acetylation events. Using solid cation exchange (SCX) after trypsin treatment, neo-N-termini generated by protease treatments can be separated from blocked, natural N-termini. The resulting N-terminal peptides can be fractionated and derivatized further to reduce sample complexity, as in the N-terminal combined fractional diagonal chromatography (N-terminal COFRADIC) protocol^90,91^, or they can be directly quantified, as in the stable-isotope protein N-terminal acetylation quantification (SILProNAQ) approach^92,93^ (Fig. 6a). The main advantage of these techniques is that they enable quantitative characterization of the in vivo Nt-acetylome and are well suited to discover NAT substrates when used as readouts for knockout or knockdown experiments. The disadvantages are mainly related to cost and labor, as these techniques require extensive fractionation steps, significant mass spectrometer instrument time, and specialized expertise to perform data analysis (Table 2). COFRADIC has long been the benchmark for in vivo Nt-acetylation measurements and has been successfully used to estimate changes in the acetylation stoichiometry of individual N-termini, including measurements of partial acetylation. The SILProNAQ method is relatively new, and so far, it has not been used for partial acetylation measurements^58,92^, although it in principle is capable of performing these measurements. COFRADIC generally has more coverage (between 583 and 2624 identified unique N-termini in recent datasets^6,47,53^ compared to between 270 and 638 N-termini for SILProNAQ^58,92^), which may be attributed to more extensive fractionation in the COFRADIC protocol than in SILProNAQ. While this greater coverage gives COFRADIC an edge compared to SILProNAQ, some of this gap is expected to be filled by faster and more sensitive mass spectrometers.

**Table 2:** Overview of Nt-acetylation experimental techniques

	COFRADIC	SILProNAQ	In vitro peptide library	In vitro NAT assay	NBD-Cl assay
Detects native acetylations?	Yes	Yes	No	No	Yes (in aggregate)
Substrate discovery?	Yes	Yes	Yes	No	No
Proteome scale or single substrate?	Proteome scale	Proteome scale	Many substrates, but it uses artificial N-termini	Single substrate	Proteome scale, but it does not distinguish individual substrates
Cost	$$$$	$$$	$$	$	$
Can identify partially Nt-acetylated proteins?	Yes	Yes	No	No	No
Applicable to patient samples / model organism tissues?	Yes	Yes	No	No	Yes
Main advantages	Powerful, proteome-scale quantitation and substrate discovery, detects native acetylations, superior coverage	Powerful, proteome-scale quantitation and substrate discovery, detects native acetylations, less extensive fractionation compared to COFRADIC	Quantitative and unbiased substrate specificity discovery	Determines kinetic parameters, can be used for inhibitor studies, several readout options available (DTNB, HPLC, and ^14^C-Ac-CoA), suitable for high-throughput screening	Quick and simple, useful for a wide range of samples, detects native acetylations (in aggregate)
Main drawbacks	Expensive and time-consuming, extensive fractionation compared to SILProNAQ	Expensive and time-consuming, low coverage compared to COFRADIC	Kinetics not possible	Single substrate only, relatively insensitive (depending on readout method)	No substrate discrimination
Key references	^90,91^	^92,93^	^43,49^	^94–96^	^97,98^

Another method for unbiased substrate discovery for known or suspected NATs is the in vitro peptide library approach (Fig. 6b). Any proteome sample can be used as an input material. The purpose is to acquire, upon trypsin treatment, peptides with a broad spectrum of N-termini. These peptides are obtained in the same manner as they are in COFRADIC/SILProNAQ, but instead of discarding the internal peptides resulting from proteolysis at the SCX step, these peptides are retained and used as substrates in an in vitro NAT assay. The resulting in vitro enzymatically Nt-acetylated peptides are then analyzed by LC/MS, and the sequence features of the substrates are obtained, showing good agreement with the specificity data obtained through focused NAT assays for synthetic peptides with known sequences^43,49^. To obtain kinetic parameters and implement inhibitor testing, the method of choice is in vitro NAT assays using recombinant NAT enzymes and synthetic peptides with known sequences (Table 2). There are several readout variations possible for such assays, such as the 5-5′-dithiobis-(2-nitrobenzoic acid) (DTNB)^94^, HPLC^95^ or ^14^C-acetyl-CoA^96^ method.

The 4-chloro-7-nitrobenzofurazan (NBD-Cl) assay is a potentially versatile method for quantifying the global loss of N-terminal acetylation as a response to pharmacological or genetic perturbation of NAT enzymes^97,98^. This assay relies on treating native proteins with the fluorescent dye NBD-Cl, which binds to primary amines and fluoresces upon binding. For the assay to be specific for the N-terminal amine group, careful control of the pH in the reaction buffer is required, as the dye will conjugate to N-termini but not to lysine amines at neutral pH.

In conclusion, the future of the Nt-acetylation field looks bright. New methods take advantage of faster and more sensitive mass spectrometers, leading to increased quantitative coverage of in vivo Nt-acetylation sites and how they change with genetic and pharmacological manipulation. Animal models with KO or mutant alleles for NAT-encoding genes provide troves of data on the organismal impact of a process that is becoming more clearly described, but the significance of this process still is poorly understood.
